# Virus Entry by Endocytosis

**DOI:** 10.1155/2013/469538

**Published:** 2013-04-21

**Authors:** Anthony V. Nicola, Hector C. Aguilar, Jason Mercer, Brent Ryckman, Christopher M. Wiethoff

**Affiliations:** ^1^Department of Veterinary Microbiology and Pathology, Washington State University, Pullman, WA 99163, USA; ^2^Paul G. Allen School for Global Animal Health, Washington State University, Pullman, WA 99163, USA; ^3^Institute of Biochemistry, ETH Zurich, 8093 Zurich, Switzerland; ^4^Division of Biological Sciences, University of Montana, Missoula, MT 59812, USA; ^5^Department of Microbiology and Immunology, Loyola University Chicago Stritch School of Medicine, Maywood, IL 60153, USA

Virus entry is the process by which the incoming viral particle gains access to the host cell. Investigation of viral entry reveals novel targets for interfering with the virus before it can commandeer the host cell machinery for replication. Viruses use virally encoded and cellular factors to overcome multiple obstacles for successful entry, allowing for the delivery of the viral genome to the preferred subcellular site of viral replication. Virus-receptor interactions can play multiple roles in viral entry, including cell attachment, endocytosis, membrane fusion, and molecular signaling. Endocytosis is a fundamental cellular activity that is utilized by the majority of animal virus families to introduce their genetic material to the cell interior. 

The different pathways subjugated by viruses include clathrin-mediated endocytosis, caveolae, macropinocytosis, and nonclathrin, noncaveolae routes. Endocytic processes offer several advantages to the incoming virus, including transit through the cell periphery, which can contain a thick layer of cortical actin. The acidic environment of an endosomal compartment is often needed to mediate penetration into the cytoplasm. This special issue on viral entry via endocytosis compiles five topical review articles on several diverse animal viruses.

Two papers in this special issue review the entry of members of the nonenveloped picornavirus family. R. Fuchs and D. Blaas focus on the entry of types A, B, and C human rhinoviruses, a group of more than 100 distinct serotypes for which there are no vaccines. For nonenveloped viruses, delivery of genetic cargo to the cytoplasm can occur by pore formation or rupture of an endocytic membrane. Continuing on this theme, P. Merilahti et al. review the cellular entry pathways utilized by three additional human picornaviruses: coxsackievirus A9, echovirus 9, and parechovirus 1. These viruses share the common ability to bind to host cell integrins during viral entry.

Herpesviral entry is a complex interplay of multiple viral envelope proteins and multiple cellular factors. In the third paper, T. Maeki and Y. Mori present a review of the cell entry of two closely related betaherpesviruses, human herpesvirus-(HHV-)6A and HHV-6B. Interactions of both HHV-6 species with the host cell receptor CD46 are considered. These viruses contain distinct glycoprotein complexes comprised of gH-gL and additional proteins. This is a characteristic shared by other human herpesviruses, such as human cytomegalovirus and Epstein-Barr virus. HHV-6A and HHV-6B both contain complexes of gH/gL/gO and gH/gL/gQ1/gQ2. 

In another paper, E. Rumschlag-Booms and L. Rong review the host cell entry of the global respiratory pathogen influenza A virus. Sialic acid moieties are critical for influenza pathogenesis, serving as binding receptors for entry and as determinants of tropism. Focus is on the genetics of influenza A viruses that contribute to entry tropism and on developing anti-influenza entry therapeutics and their mechanism of action. Endocytosis is an important pathway for the entry of several retroviruses. In a paper of this special issue, Y. Kubo et al. review the role of endosomal acidification and cathepsin proteases in retroviral entry with an emphasis on murine leukemia viruses (MLVs) and human immunodeficiency virus (HIV). Reports of the pH-dependent entry of CD4-dependent and -independent HIVs are also discussed.

*Anthony V. Nicola* *Hector C. Aguilar* *Jason Mercer* *Brent Ryckman* *Christopher M. Wiethoff*

